# A Role of PI3K/Akt Signaling in Oocyte Maturation and Early Embryo Development

**DOI:** 10.3390/cells12141830

**Published:** 2023-07-12

**Authors:** Jaroslav Kalous, Daria Aleshkina, Martin Anger

**Affiliations:** 1 Institute of Animal Physiology and Genetics, Academy of Sciences of the Czech Republic, 277 21 Libechov, Czech Republic; aleshkina@iapg.cas.cz (D.A.); anger@iapg.cas.cz (M.A.); 2 Department of Cell Biology, Faculty of Science, Charles University, Albertov 6, 128 00 Praha, Czech Republic

**Keywords:** Akt kinase, mTORC1, mitosis, meiosis, spindle, oocyte, early embryo, mRNA translation

## Abstract

A serine/threonine-specific protein kinase B (PKB), also known as Akt, is a key factor in the phosphoinositide 3-kinase (PI3K)/Akt signaling pathway that regulates cell survival, metabolism and proliferation. Akt phosphorylates many downstream specific substrates, which subsequently control the nuclear envelope breakdown (NEBD), centrosome maturation, spindle assembly, chromosome segregation, and cytokinesis. In vertebrates, Akt is also an important player during oogenesis and preimplantation development. In the signaling pathways regulating mRNA translation, Akt is involved in the control of mammalian target of rapamycin complex 1 (mTORC1) and thereby regulates the activity of a translational repressor, the eukaryotic initiation factor 4E (eIF4E) binding protein 1 (4E-BP1). In this review, we summarize the functions of Akt in mitosis, meiosis and early embryonic development. Additionally, the role of Akt in the regulation of mRNA translation is addressed with respect to the significance of this process during early development.

## 1. Introduction

Female germ cells and developing embryos are unique in many aspects, including their life cycle, cell cycle regulation and transcriptional silencing. In mammals, the primordial germ cells appear early during embryonic development. After migration to the gonadal ridges, which will eventually develop into gonads, cells increase their numbers by undergoing multiple mitotic divisions. Then, after entering meiosis while still in utero, they become arrested in the prophase of the first meiotic division until puberty, after which they are gradually recruited for further development. The key step, essential for oocyte development, is an interaction with cells of mesodermal origin within the developing gonads, which will lead into formation of the follicles. After puberty, when the follicle-stimulating hormone (FSH)/luteinizing hormone (LH) surges gradually stimulate their further development, primordial follicles, together with oocytes they surround, are recruited for growth. During this period, oocytes dramatically increase their size and eventually acquire competence to resume meiosis, after which they undergo two consecutive chromosomal divisions, without DNA replication in between, resulting in haploid cells, which are ready for fertilization. Coincidentally with reaching their full size, oocytes become transcriptionally inactive [1]. The completion of meiosis, fertilization and early embryonic development are therefore dependent on regulated translation of maternally synthetized mRNAs, accumulated during oocyte growth [2,3]. Although the maternal RNAs are highly stable in germinal vesicle (GV) stage oocytes, over 90% of these mRNAs are degraded soon after fertilization during the first mitotic divisions [4] and the future of the embryo depends on successful activation of its genome. Considering the unique life cycle of oocytes and also transcription independent control of meiosis, fertilization and early cleavage cycles of developing embryos, it is clear that pathways involved in translation and cell cycle control, including the phosphoinositide-3-kinase (PI3K)/Akt signaling pathway, are of paramount importance [5]. 

## 2. The Protein Kinase Akt

The serine/threonine protein kinase Akt (v-akt murine thymoma viral oncogene homolog) is a component of the phosphoinositide 3-kinase (PI3K)/Akt signaling cascade that plays a critical role in a variety of cellular processes, including cell cycle progression, glucose metabolism, transcription, cellular growth, embryogenesis, as well as angiogenesis [6,7,8]. The Akt kinase family is comprised of three highly homologous isoforms: Akt1, Akt2 and Akt3, which are closely related and consist of a conserved N-terminal pleckstrin homology (PH) domain, a central catalytic domain, and a C-terminal regulatory hydrophobic motif (HM) [9]. The expression of Akt isoforms is tissue-dependent; whereas the Akt1 and Akt2 are expressed in multiple tissues, the expression of Akt3 is mainly restricted to the brain [10]. The activation of Akt is initiated by its recruitment to the plasma membrane through the binding of the PH domain of Akt to the phosphatidylinositol-3,4,5-trisphosphate (PIP3). Subsequently, it requires phosphorylation by the PI3K-dependent kinase 1 (PDK1) at the threonine 308 (Thr308) residue within the activation T-loop of the catalytic domain and at serine 473 (Ser473) residue within the carboxyl terminal hydrophobic domain. The phosphorylation at Ser473 is performed by the mammalian target of rapamycin complex 2 (mTORC2) [11,12]. Akt becomes fully activated only when it is phosphorylated at both the Thr308 and Ser473 residues [13]. 

Activated Akt dissociates from the plasma membrane and it is translocated to both the cytosol and nucleus, where many of its substrates are located [14,15]. The downstream effect of Akt activity is mediated through the serine and/or threonine phosphorylation of specific substrates involved in a range of cellular and physiological processes [16,17].

Inactivation of the PI3K/Akt pathway is mainly accomplished by phosphatase and tensin homologue deleted on chromosome 10 (PTEN), which exhibits dual lipid and protein phosphatase activity [18]. PTEN specifically catalyzes dephosphorylation of the 3′ phosphate of the inositol ring in phospholipid PIP3, resulting in formation of the bisphosphate product phospholipid PIP and inhibition of Akt activity [19]. On the other hand, Akt is also inactivated directly by dephosphorylation of the pT308 residue by protein phosphatase 2A (PP2A), dephosphorylation of the pSer473 residue is accomplished by the PH domain leucine-rich repeat protein phosphatases (PHLPP) [20,21]. 

## 3. Role of Akt in Cell Cycle Control 

### 3.1. Akt Is Involved in Regulation of Mitosis 

The PI3K/Akt signaling pathway is involved in the control of cell cycle progression as well as of cell proliferation and survival [22]. Akt regulates multiple processes and pathways in each cell cycle stage. One of the best known examples is the activation of cyclin-dependent kinases (CDKs), the serine threonine kinases that play principal roles in the control of cell division [23,24,25]. During the G1 phase, Akt/mTOR has stimulatory effect on cell cycle progression by promoting the expression of cyclin D1, cyclin-dependent kinase 4 (CDK4) and phosphatase CDC25A [26]. At the G1/S transition, Akt promotes the phosphorylation and inactivation of p21WAF1 and p27kip1, the inhibitors of cyclin-dependent kinase 2 (CDK2) [27,28] (Table 1).

A significantly higher Akt activity suggests a specific role of this kinase during the transition from S to G2 phase [29,33]. The activation of CDK1, a catalytic subunit of the M phase-promoting factor (MPF), is essential for cell cycle progression through the S/G2 and G2/M phase in both meiosis and mitosis [34,35]. In the G2/M phase, Akt regulates the cell cycle progression through the direct phosphorylation of CDK1 activators and inhibitors, demonstrating the important role of PI3K/AKT pathway in promoting cell division [23,24]. Specifically, CDK1 at this point is inhibited by Wee1 and Myt1 kinases, which phosphorylate Thr14 and Tyr15 residues within CDK1 ATP binding pocket and when cells are ready for G2/M transition, Akt by phosphorylation inhibits both Wee1 and Myt1 kinases [36]. Akt also phosphorylates and activates the CDC25 phosphatase, which is important for removal of CDK1 Thr14 and Tyr15 phosphorylation, previously facilitated by Wee1 and Myt1, enabling the activation of CDK1 during the G2/M transition [24]. Akt is involved in the control of the mitotic spindle checkpoint [30], localizes to the spindle poles [37], affects the integrity and composition of mitotic centrosomes [31], and participates on the regulation of cytokinesis [32].

### 3.2. Akt Affects Progression of Meiosis

In the developing ovary, Akt promotes the proliferation of primordial germ cells [38]. After entering meiosis, mammalian oocytes are arrested in the prophase of the first meiotic division until resumption of meiosis. During the in vitro maturation (IVM) of mammalian cumulus-oocyte complexes (COCs), Akt is present in both oocytes and cumulus cells (CCs), and its activity is required for the regulation of meiotic progression [39,40,41] (Table 2).

**Figure 1:**
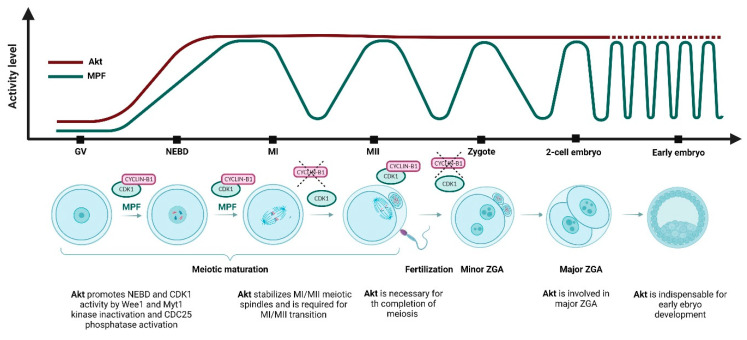
Akt and maturation-promoting factor (MPF) activity during meiotic maturation, oocyte-to-embryo transition and early embryo development. During the first meiotic arrest at the germinal vesicle (GV) stage, Akt is inactivated. During germinal vesicle breakdown (GVBD), MPF and Akt activity increases. At meiosis I (MI) stage, Akt activity is remains stable and activity of MPF reaches maximum. At the MI/meiosis II (MII) transition, cyclin B is destructed by the anaphase promoting complex/cyclosome (APC/C), MPF activity is reduced, and the first polar body is extruded. The second metaphase plate is formed at the MII stage, and meiosis is stopped in the second meiotic arrest. Akt activity is maintained after the first polar body extrusion, MPF activity is restored to the MI levels, and the oocyte awaits fertilization. After fertilization, MPF activity is downregulated, Akt activity persists in the zygote, when the second polar body is extruded, and both male and female pronuclei are formed. MPF activity is restored before the first mitotic division. At the 2-cell embryonic stage, the first mitotic division is completed, Akt remains activated and MPF activity declines. Akt is involved in the regulation of zygotic genome activation (ZGA) which occurs in mouse embryo at the 2-cell stage. During the early embryo development, the Akt activity remains at high levels and MPF activity is cyclically downregulated and restored at each subsequent mitotic division.

In the fully grown oocytes residing in the antral ovarian follicles, the Akt activity is at very low level [39,40]. In some species, its activity is however required during resumption of meiosis. In mouse oocytes for example, the phosphorylation of Akt by the upstream kinases is necessary to promote the resumption of meiosis under both in vivo and in vitro conditions [42,43,44]. Similarly to the somatic cells, in oocytes Akt has been reported to be involved in CDK1 activation and induction of germinal vesicle breakdown (GVBD); equivalent to nuclear envelope breakdown or NEBD in somatic cells. For its full activity during GVBD, Akt requires the phosphorylation of both T308 and S473 residues [43]. This crucial role of Akt in CDK1 activation and GVBD induction was also reported in starfish and zebrafish oocytes [45,46,52]. In contrast to the mouse and starfish, the Akt activity seems to be dispensable during GVBD in porcine oocytes. Another meiotic event, during which the Akt activity is required, is the transition from meiosis I to meiosis II. The role of Akt during this process seems to be generally conserved, as it was shown in bovine [39,47], mouse [43,48] *Xenopus* [49] and porcine oocytes [40] (Table 2; Figure 1). 

Spindle assembly during meiosis is unique. In contrast to mitosis in somatic cells, and also to sperm meiosis, in which the spindle assembly is driven by centrosomes, in oocytes the assembly of the spindle in both meiotic divisions is acentrosomal and requires instead the clustering of microtubule-organizing centers (MTOCs) [53,54,55]. The centrosomes are lost during oocyte development [56] and this fundamental difference between spindle assembly in somatic cells and oocytes might contribute to the higher incidence of chromosome segregation errors in the latter. The localization pattern on meiotic spindle indicates an important role of Akt in the assembly and stabilization of this structure [43,48,50]. During oocyte meiosis, Akt becomes phosphorylated at either Ser473 (pS473-Akt) or Thr308 (pT308-Akt) residues, which has consequences for the diversity of Akt functions. It also affects the localization pattern on meiotic spindle. Whereas pT308-Akt localization is restricted to the spindle poles, pS473-Akt is detected along the spindle microtubules [43,48,50]. And for correct spindle assembly in meiosis II, Akt carrying simultaneously phosphorylation of both Ser473 and Thr308 residues, is required [48]. 

In addition, Akt is an important player in the regulation of apoptosis, in somatic cells Akt prevents the translocation of the BAX protein to the mitochondria to start apoptosis [57]. Akt is also involved in the control of apoptosis during meiosis, the elevation of Akt activity in matured oocytes led to an enhanced expression of genes involved in cell signaling and proliferation, and to a decreased expressions of pro-apoptotic genes such as *BAX*, *BCL2* and *caspase-3* [58]. It has been suggested that Akt could be involved in the regulation of the expression, activity and localization of pro-apoptotic proteins in oocytes and, moreover, AKT could stabilize endogenous apoptosis inhibitors [59].

Overall, Akt plays a critical role in regulating various aspects of meiosis, including entry into meiosis, progression through the MI to MII stage, and apoptosis. The dysregulation of Akt activity during oocyte maturation can lead to defects in meiosis and can result in infertility or birth defects.

## 4. Role of Akt in Oogenesis and Folliculogenesis 

### 4.1. Akt Regulates Follicle Development

The Akt signaling pathway is an important regulator of ovarian functions, such as the survival, proliferation, and differentiation of granulosa cells (GCs) [60]. Akt also regulates quiescence and the activation of primordial follicles [61]. The development of ovarian follicles begins with the proliferation of GCs, which are the predominant somatic cell type of the ovarian follicle and are involved in steroidogenesis and folliculogenesis [62]. During the growth and development of the ovarian follicle, the undifferentiated GCs are present from primordial to preantral follicles [63]. In antral follicles, GCs differentiate into CCs and mural granulosa cells (MGCs), CCs surround the oocyte and MGCs form a layer that is firmly attached to the inner wall of the follicle. During folliculogenesis in the human ovary, Akt has been detected in oocytes, in MGCs and in the thecal cells of primordial follicles, in growing follicles, and in the luteal cells [64]. The results on mouse knock-out models revealed the importance of Akt in oocyte growth and in the expression of cell-cycle regulators. The fertility of Akt1^−/−^ female mice was reduced due to the altered follicular development and abnormal oocyte growth [65]. In the Akt1^−/−^ ovaries, there was a reduced expression of cell-cycle regulators cyclin D1 and cyclin D3, and the expression of the survival factor KIT ligand and anti-apoptotic factor BCL2 like 1 was also decreased [65]. In the cultured ovine preantral follicles, activation of the PI3K/AKT pathway led to a promotion of primordial follicle activation and cell proliferation, and resulted in a reduction of DNA fragmentation [66]. 

### 4.2. Akt Promotes Survival of GCs 

MGCs, which form an inner layer of the ovarian follicle, play a crucial role in the development of oocytes as they produce factors essential for oocyte growth and folliculogenesis [67]. The activity of the PI3K/Akt pathway is necessary for the induction of numerous critical genes that mark the fully differentiated preovulatory MGCs [68,69]. The PI3K/Akt pathway in in vitro cultured GCs can be activated by insulin-like growth factor 1 (IGF1) via the type-I IGF receptor (IGF1R), indicating that the IGF1-regulation of the PI3K/Akt pathway activity plays role in the control of cell cycle progression as well as in the promotion of GCs survival [70,71]. The IGF1-activated PI3K/Akt pathway protects GCs from apoptosis, and this protective effect can only occur when progression from the G1 to S phase of the cell cycle, regulated by the PI3K/Akt pathway, is not disrupted [72]. Mechanisms leading to the regulation of Akt phosphorylation induce changes in the proliferation and differentiation of MGCs cells during the growth of ovarian follicles [64]. The promoting effect of the PI3K/Akt signaling pathway activity on cell survival within the granulosa layer is important for the development of the preovulatory ovarian follicle [73]. 

### 4.3. Activity of Akt in CCs

In mammals, CCs surrounding the oocyte play an important role in oocyte growth, meiotic maturation, ovulation and fertilization [74]. The inner layer of CCs known as the corona radiata, and the oocyte together with the surrounding CCs form the COC [75]. The activity of the PI3k/Akt pathway in CCs has at least two important roles during the meiotic maturation of oocytes [76]. Firstly, the low level of Akt activity in CCs is essential for the establishment of meiotic arrest. And secondly, Akt activation in CCs is linked to the induction of gonadotropin-stimulated meiotic resumption.

CCs regulate oocyte development and meiotic maturation by generating paracrine factors and, moreover, CCs control the resumption of meiosis and formation of the oocyte cytoskeleton [77]. Activated PI3K/Akt in CCs ensures the transduction of pro-survival signals essential for oocyte development [63]. Specific CCs genes strictly associated with the oocyte developmental competence are regulated by the PI3K/Akt pathway [78].

### 4.4. Akt Regulates Signaling between CCs and Oocytes

In developing mammalian follicles, CCs transport nutrients such as amino acids and substrates for energy production to the oocytes via cytoplasmic processes known as transzonal projections (TZPs), which originate from CCs and penetrate the zona pellucida, the outer layer of the oocyte [79,80,81]. Terminally, TZPs form heterologous gap junctions with cytoplasmic membrane of oocyte. Gap junctions are intercellular channels that permit the direct transfer of ions and small molecules (<1 kDa) between adjacent cells [82]. The close relationship of oocytes and CCs is crucial for the regulation of oocyte growth and development. The bidirectional communication between the oocyte and CCs via heterologous gap junctions is essential for the formation of a developmentally competent oocyte, which can be fertilized, and which is also able to support subsequent embryonic development [83]. The PI3K/Akt pathway plays an essential role in the communication between the developing oocyte and CCs [84]. Gap junction channels are composed of connexins (Cx), and Cx proteins enable the communication between the oocyte and accompanying CCs [85]. In somatic cells, Akt mediates the phosphorylation of Cx43 in gap junctions itself [86]. In COCs, the activity of the PI3K/Akt signaling pathway is closely associated with the progression of meiosis via the regulation of CX43 phosphorylation in CCs [87,88]. 

### 4.5. Role of Akt in Expansion of CCs

The PI3K/Akt pathway not only promotes the nuclear maturation of oocytes, but also supports expansion of the oocyte cumulus layer, a process that is important for the maturation and fertilization of oocytes [75]. In response to LH surge, CCs surrounding the oocyte synthesize a large amount of high molecular weight glycosaminoglycan hyaluronan (HA), a major component of the extracellular matrix (ECM) that plays multiple roles during and after fertilization [89]. The cross linking of HA with tumor necrosis alpha-induced protein 6 (TNFAIP6), inter-alpha-trypsin inhibitors (IαI), and pentraxin 3 (PTX3) is a pre-requisite for the appropriate formation of ECM, essential for oocyte ovulation and fertilization [89,90,91]. HA synthesis in the FSH-stimulated COCs and retention of HA in the cumulus ECM are PI3K/Akt dependent [92]. Inhibition of the PI3K/Akt pathway dramatically reduced the expression levels of hyaluronan synthase 2 (HAS2) and TNFAIP6, the key enzymes involved in the production and stabilization of HA in the expanding cumulus, indicating that the activity of Akt is essential for the expression of genes involved in the expansion of the oocyte cumulus [93,94]. It has been proposed that activation of a downstream Akt effector, the mammalian target of rapamycin (mTOR), in the oocyte cumulus cells is essential for the production of functionally competent matured oocytes [95].

## 5. The Role of Akt in mRNA Translation

### 5.1. Akt Regulates mTOR Activity during Mitosis 

One of the key downstream targets of Akt is the mTOR pathway, which plays a critical role in the regulation of mRNA translation, and the link between the PI3K/AKT and mTOR pathway is essential for oocyte meiotic maturation [61]. The mTOR pathway is activated by a variety of signals, including growth factors, nutrients, and energy status. MTOR, a serine-threonine kinase, is a component of mTOR complex 1 (mTORC1) and mTORC2, two cellular complexes that have distinct functions and regulation [96,97]. MTORC1 controls the translation of several proteins that are important for growth and cell cycle progression [98,99]. Akt directly phosphorylates and activates mTORC1, which in turn stimulates the phosphorylation of key downstream targets, including the mTORC1 effectors p70 ribosomal protein S6 kinase (p70S6K), which phosphorylates the eukaryotic translation initiation factor 4B (eIF4B) and is essential for the translation of 40s ribosomal S6 protein, and 4E-BP1, which regulates cell proliferation via the control of the cap-dependent translation and acts as a translational repressor [100,101,102]. The phosphorylation of p70S6K and 4E-BP1 mediates the transduction of mitogen and nutrient signals to stimulate translation [103]. 

**Figure 2:**
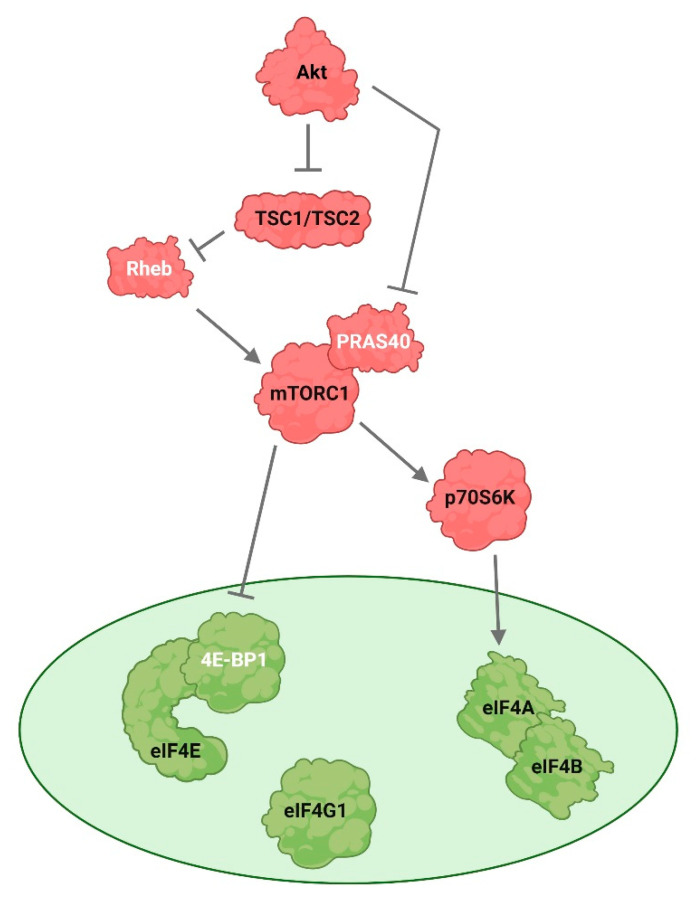
Akt regulates mRNA translation. Akt directly activates mammalian target of rapamycin complex 1 (mTORC1) by phosphorylating and inactivating proline-rich Akt substrate 40 kDa (PRAS40), a protein that is associated with mTORC1. Further, Akt also activates mTORC1 indirectly by phosphorylating and inactivating the tuberous sclerosis complex 1 (TSC1) and tuberous sclerosis complex 2 (TCS2). TSC1/TSC2 functional complex acts as a key upstream negative regulator of mTORC1 activity. TSC2 has a role as a GTPase-activating protein (GAP) which inactivates an essential mTORC1 activator, the RAS homologue enriched in brain (Rheb). MTORC1 phosphorylates and activates the 70-kDa ribosomal protein S6 kinase (p70S6K), which phosphorylates the eukaryotic translation initiation factor 4B (eIF4B). The eukaryotic translation initiation factor 4E (eIF4E)-binding protein 1 (4E-BP1), phosphorylated and activated by mTORC1, is released from eIF4E and the assembly of the eIF4F complex is enabled. Factors involved in cap-dependent translation initiation are depicted in green, the upstream factors in red. Stimulatory modification is depicted as an arrow, inhibitory modification as a blunt end line.

Akt-activated mTORC1 pathway promotes the translation of mRNAs involved in cell cycle progression, such as cyclins and CDKs [61]. Hypophosphorylated 4E-BP1 prevents eIF4E from associating with eIF4G. Upon phosphorylation by Akt and mTORC1, 4E-BP1 dissociates from eIF4E, and this event enables assembly of the eIF4F complex [104,105]. The deregulation of protein synthesis downstream of mTORC1 at the level of 4E-BP1/eIF4E plays a central role in tumor formation, and 4E-BP1/eIF4E transfers the effect of oncogenic Akt signaling on mRNA translation, cell growth, and tumor progression [106]. Akt directly activates mTORC1 by phosphorylating the proline-rich Akt substrate 40 kDa (PRAS40), a protein that associates with mTORC1 and regulates mTORC1 kinase activity by the direct inhibition of substrate binding [107,108]. Akt also activates mTORC1 indirectly by phosphorylating and inactivating two tumor suppressor proteins, tuberous sclerosis complex 1 (TSC1), also known as tuberin, and tuberous sclerosis complex 2 (TCS2), also known as hamartin [109,110]. TSC2 has a role as a GTPase-activating protein (GAP) which inactivates an essential mTORC1 activator, the RAS homologue enriched in the brain (Rheb) [111]. TSC1 and TSC2 form a functional complex that acts as a key upstream negative regulator of mTORC1 kinase activity and exerts its effects through mTORC1 to regulate the activity of p70S6K and 4E-BP1 [110] (Figure 2). 

Additionally, Akt can indirectly regulate mRNA translation by affecting the activity of other translation factors, such as eukaryotic initiation factor 2B (eIF2B) and eukaryotic elongation factor 2 (eEF2), which are involved in the initiation and elongation phases of mRNA translation, respectively [112,113].

### 5.2. Akt Affects mTORC1 Activity during Oocyte Meiosis

MTORC1 is expressed in all stages of oocytes development, suggesting its fundamental role in regulation of oocyte meiosis and early embryonic development [114]. In oocytes, mTORC1 acts as a downstream Akt effector, and mTORC1 activation in CCs is essential for the production of functionally competent mature oocytes [95].

**Figure 3:**
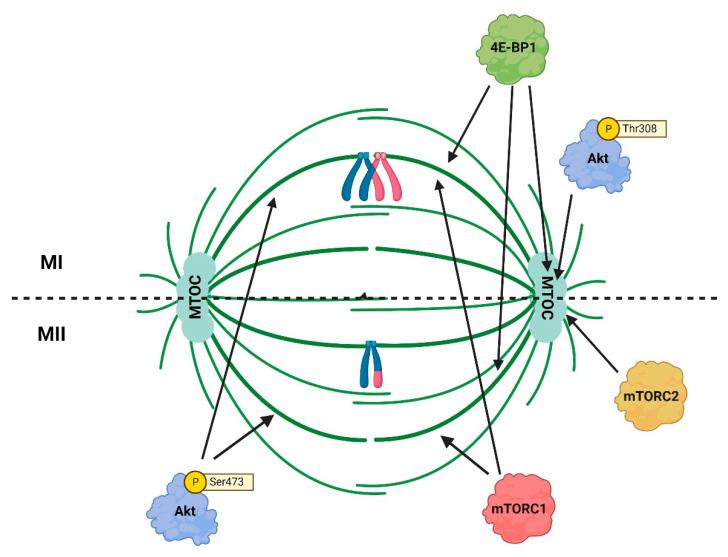
Co-localization of Akt with 4E/BP1 and mTORC1/mTORC2 at the meiosis I (MI) and meiosis II (MII) spindles. The p473-Akt and mTORC1 are distributed along microtubules, the p308-Akt and mTORC2 are localized at spindle poles where microtubule-organizing centers (MTOCs) are positioned. 4E-BP1 is detected along microtubules and at spindle poles.

In mammalian oocytes, the temporal and spatial control of translation is regulated via an mTORC1-eIF4F pathway [115]. The incorporation of eIF4E into the pathway required for the initiation of translation is regulated by its phosphorylation in oocytes as well as through binding of inhibitory proteins [116]. In GV stage oocytes, mTORC1 is localized to the cytoplasm, at GVBD it is distributed around chromosomes, and at M-phase it is localized on the meiotic spindles in the vicinity of the chromosomes [117]. During meiotic maturation of mouse oocytes phosphorylated Akt localizes to meiotic spindle, and this localization overlaps with mTORC1 localization [43,48]. It is tempting to speculate that Akt regulates a localized translation of specific mRNAs, necessary for spindle assembly, via co-localization with mTORC1 on the spindle (Figure 3).

Although phosphorylated Akt is an upstream activator of mTORC1 in somatic cells, it has been suggested that during the meiosis of mammalian oocytes, the Akt pathway is not sufficient for full mTORC1 activation, which is likely to be mediated by CDK1 instead [118]. However, downregulation of the AKT/mTORC1 pathway and its downstream signaling cascades during IVM reduces the quality and developmental potential of porcine and bovine oocytes [58,119]. In MII oocytes, phosphorylated Akt is localized to the MII spindle along with ribosomal protein S6 (RPS6) and 4E-BP1 [48,118]. This suggests that Akt, localized to the MII spindle, regulates the activity of the mTORC1 pathway components.

## 6. Akt in Zygotic Transition and Early Embryo Development

After fertilization, the PI3K/AKT pathway becomes activated by autocrine trophic ligands, and the activity of this pathway is essential for early embryo development [120,121]. In the 2-cell mouse embryo, active mTORC1 is required for 4E-BP1 phosphorylation and eIF4E activation, a prerequisite for the initiation of mRNA translation [122]. The maternally provided eIF4E is able to support mouse embryo up to the two- to four-cell stage, after which a newly expressed eIF4E from the embryonic genome is required, marking the important switch from maternal to embryonic control of translation during development [122]. 

MTORC1 is activated under favorable conditions such as the availability of amino acids (AAs), growth factors, and intracellular ATP. The importance of the mTORC1 pathway for promoting normal preimplantation development can be demonstrated on embryos cultured in vitro. Although in mouse the simple culture media without amino acids are able to support embryo development to the blastocyst stage, the preimplantation embryo viability and developmental potential could be improved by supplementing the culture media with essential and nonessential amino acids [123,124]. In the bovine and human embryos cultured in the absence of AAs, the phosphorylation of Akt on Ser473 is decreased, together with declined of mTORC1 signaling, resulting in compromised preimplantation development, partially restorable by addition of AAs [125]. The PI3K/Akt signaling pathway is also important for the normal development of the early embryo to the blastocyst stage [126] (Table 3).

In the early drosophila embryos, Akt regulates centrosome migration, mitotic spindle orientation, and promotes proper spindle morphology [133]. In zygote, pSer473-Akt localizes to both male and female pronuclei and similarly to somatic cells plays important role during the mitotic entry [127,128]. In 2-cell embryos, the pSer473-Akt also shows nuclear localization [129]. The nuclear localization of pSer473-Akt during major zygotic genome activation (ZGA), and the 2-cell embryos arrest induced by the specific Akt inhibition indicate that Akt is possibly involved in the major ZGA in 2-cell mouse embryos [130]. Moreover, the inhibition of Akt compromised the development of mouse embryos to the blastocyst stage, suggesting that Akt activity has a significant effect on normal blastocyst development [120,132]. It has been proposed that the regulation of blastomere proliferation in preimplantation mouse embryos is based on Akt activity [128]. Activated Akt is essential for mouse blastocyst formation and for expression of the trophectoderm marker Cdx2, indicating that Akt may be indispensable for the first cell lineage differentiation in the mouse early embryo [134]. The importance of Akt signaling for the developmental competence of the early embryo was revealed by Akt inhibition that resulted in a reduction of early embryo cleavage and compromised embryo development to the 8- to 16-cell and blastocyst stages [131]. The data from *Xenopus* and starfish early embryos suggest that Akt is involved in the regulation of the mitotic G2 to M phase transition through the activation of M-phase promoting factor (MPF) [36,46]. In summary, Akt plays a critical role in regulating mRNA translation, embryonic development, and cell survival during the early stages of embryogenesis.

## 7. Conclusions

Akt plays an important role in regulating various aspects of cell cycle, it is involved in the control of key points in meiosis and mitosis. In oocytes, Akt supports the entry into meiosis and transition from meiosis I to meiosis II. Akt is also essential for embryonic development after fertilization. Dysregulation of Akt activity during the meiotic maturation of oocytes can lead to defects in meiosis and impair preimplantation development resulting in infertility or birth defects. The summarized data indicate that the gene expression and activity of the PI3K/AKT pathway can be possibly used as a predictive marker for the developmental competence of oocytes and successful embryo implantation. Detailed identification of PI3K/Akt/mTOR downstream factors that promote developmental competence of oocytes could be of importance for assisted reproduction. 

## Figures and Tables

**Table 1:** Role of Akt in regulation of mitosis.

Cell Cycle Stage	Akt Role in Mitosis	References
G1 phase	Akt/mTOR stimulates expression of cyclin D1, CDK4 and CDC25A, that are involved in cell growth and proliferation	[26]
G1/S	Akt promotes inactivation of p21WAF1 and p27kip1, the CDK2 inhibitors	[27,28]
S/G2	Enhanced Akt activity indicates a role for Akt in the S/G2 transition	[29]
G2/M	Akt regulates cell cycle progression by direct phosphorylation and inactivation of Wee1 and Myt1 kinases and activates the CDC25 phosphatase	[23,24,25]
M-phase	Akt is involved in the control of the mitotic spindle checkpoint affects the integrity and composition of mitotic centrosomes	[30,31]
Cytokinesis	Akt participates in the regulation of cytokinesis	[32]

**Table 2:** Role of Akt in meiosis progression.

Meiosis Stage	Akt Role in Meiosis	References
Prophase of 1st meiosis	Akt is involved in CDK1 activation and GVBD induction during meiosis resumption	[42,43,44,45,46]
MI/MII transition	Akt is required for the transition from meiotic metaphase I (MI) to metaphase II (MII)	[39,43,47,48,49]
MI and MII-phase	Akt participates in the formation and stabilization of the MI and MII meiotic spindles	[43,48,50]
MI and MII-phase	Akt contributes to centrosome integrity in oocytes	[43]
Meiosis completion	Akt is necessary for completion of meiosis	[47,48,51]

**Table 3:** Role of Akt in the early embryo development.

Stage of Early Embryo	Role of Akt in the Early Embryo Development	References
1-cell	Akt is essential for the entry of 1-cell mouse embryos into the first mitosis	[127,128]
2-cell, ZGA	pSer473-Akt is localized to the nuclei of 2-cell embryos, Akt is possibly involved in the major ZGA of 2-cell mouse embryos	[129,130]
8- to 16-cell	Akt is important for mouse embryo development to the 8- to 16-cells	[131]
Blastocyst	Akt is necessary for embryo development to the blastocyst stage	[120,126,128,131,132]
Early embryo	In the early *Drosophila melanogaster* embryo, Akt regulates centrosome migration, promotes mitotic spindle orientation and proper spindle morphology	[133]

## Data Availability

No new data were created.
